# Leading with a cool head and a warm heart: trait-based leadership resources linked to task performance, perceived stress, and work engagement

**DOI:** 10.1007/s12144-022-03767-8

**Published:** 2022-11-21

**Authors:** Anna Maria Dåderman, Petri Juhani Kajonius, Angela Hallberg, Sandra Skog, Åke Hellström

**Affiliations:** 1 Department of Social and Behavioural Studies, University West, Trollhättan, Sweden; 2 Department of Psychology, Lund University, Lund, Sweden; 3 Department of Psychology, Stockholm University, Stockholm, Sweden

**Keywords:** Successful leadership, Trait emotional intelligence, Personality, Coping resources for stress, Empathy and compassion, Socially desirable responding

## Abstract

Leaders of today need to achieve well in terms of task performance, perceiving low stress, and having high levels of work engagement. One may ask whether trait-based leadership resource factors can be identified and how such resource factors might relate to task performance, perceived stress, and work engagement. Our aim was to test the hypothesis, derived from Hobfoll’s motivational Conservation of Resources (COR) theory, that there are trait-based leadership resource factors, which are differentially correlated to the leaders’ task performance, perceived stress, and work engagement. Leaders (*N* = 344) aged from 23 to 65 years (*M* = 49, *SD* = 8.6; 58% women) completed an online questionnaire including measures of task performance, perceived stress, work engagement, personality traits, trait emotional intelligence, empathy, performance-related self-esteem, compassionate and rational leadership competence, and coping resources for stress. Using exploratory factor analysis, we identified four trait-based leadership resource factors. With Bonferroni adjustment, and controlling for sex, age, number of years in the current managerial position, self-deceptive enhancement, and impression management, only Rational Mastery was significantly positively correlated with task performance. Rational Mastery, Efficient Coping, and Modesty were negatively correlated with perceived stress, and all factors except Modesty, but including the fourth (Good-Heartedness) were positively correlated with work engagement. Organizations striving for sustainable work conditions should support trait-based leadership, which depends not only on a task-oriented resource such as rational mastery, but also on human-oriented resources such as efficient coping, modesty, and good-heartedness, all of them being differentially related to task performance, perceived stress, and work engagement.

**Supplementary Information:**

The online version contains supplementary material available at 10.1007/s12144-022-03767-8.

## Introduction

Working life today is characterized by rapid changes. Recent demands related to the coronavirus pandemic (COVID-19) have created an increased need for a strong, trait-based, and evidence-based leadership. Competition in stressful situations requires leaders who perform well and possess high levels of work engagement and low perceived stress. This study attempts to capture factors of trait-based leadership resources which could be useful for coping with stress, task performance and work engagement at the managerial positions. First, we explain what we mean with successful and effective leadership and define the term trait-based leadership. Then, we start from Hobfoll’s (1989) theoretical perspective on the conservation of resources, and apply it to leadership. Next, based on trait theories, we will try to identify generally valuable traits and resources that previous research has shown to be related to successful and effective leadership.

*Successful and effective leadership* is characterized by using individual resources in line with the organization’s idea and vision, focusing on goals that must be followed up (e.g., satisfied customers, good sales figures), economizing with given financial resources, obtaining new resources in competition with other actors, and keeping the organization in a good balance of risks and growth. Also, a successful leader is skilled in communicating externally and internally, and in developing and maintaining good relationship with co-workers. It is therefore obvious that the leader’s personal ability to establish and maintain networks will promote success. To this end, high levels of work engagement are required of leaders who are expected to motivate and inspire their followers by being good role models (Rahmadani et al., 2020; Schaufeli, 2021). Since task performance, low perceived stress, and high work engagement are key success factors in today’s leadership (Harms et al., 2017; Schaufeli, 2021), it is interesting to investigate whether trait-based leadership resources exist, which correlate significantly to task performance, perceived stress, and work engagement.

Historically, different perspectives on leadership have been presented, and *trait-based* perspectives of leadership are an evident perspective within leadership research (Zaccaro, 2007). Traits are habitual patterns of behavior, thoughts, and emotions that make it possible to see and describe that one person differs from others. It is evident that traits, and thereby behavior, are consistent across situations and time (Allport, 1937; Zaccaro, 2007). The variation in any trait depends on both genetic and environmental factors. A broad definition of leader traits comprises “relatively coherent and integrated patterns of personal characteristics, reflecting a range of individual differences, that foster consistent leadership effectiveness across a variety of groups and organizational situations” (Zaccaro, 2007, p. 7). According to this definition, it seems to be important to capture such complex leadership patterns or factors of traits, rather than studying these in isolation. One way is to study traits as leadership resources. In the current study, such trait-based leadership resources will be investigated in leaders from diverse organizations.

## Hobfoll’s Conservation of Resources Theory

The current study, applying Hobfoll’s Conservation of Resources Theory (COR; Hobfoll, 1989), is focused on the identification of potential trait-based leadership resources, examining them in the context of task performance, perceived stress, and work engagement, three important aspects of effective leadership. According to the COR theory (Hobfoll, 1989), resources are, among other things, personal dispositions, such as emotional intelligence and competences/skills, coping resources, self-esteem, personality traits, and empathy (Xanthopoulou et al., 2009). The COR theory considers behaviors as based on the evolutionary need to “acquire and conserve resources for survival, which is central to human behavioural genetics” (Hobfoll et al., 2018). A leader must not only survive but be engaged to master their leader role, be supportive, master their level of stress, and identify coping resources. COR theory states that people will individually define their resources – what is important and valuable to them. One such resource is the managerial position *per se*. This position has usually been achieved in hard competition; the leader has successfully convinced the organization and followers of his or her valuable traits, personality, skills, etc. The managerial position enables the leader to gain new valuable resources that he or she will retain and protect, such as higher social standing, economic stability, power, wealth, self-esteem, and knowledge. As stated by Hobfoll et al. (2018), resources are lost faster than they are gained. Since even small losses can be significant for survival, by an evolutionary principle the person must invest resources to protect resources. In practice, this may involve direct replacement of resources (e.g., using savings to cover losses) or indirect investment (e.g., increasing one’s skills to prepare for a changing environment). People who run low on resources have fewer possibilities to obtain new resources. In a context of resource loss, the existing resources increase in value and gaining new resources increases in importance.

In the context of leadership, and relevantly for our study which examines a number of trait-based leadership resources together, the COR theory uses two metaphors: *resource caravans* and *resource caravan passageways*. The metaphor of resource caravans states that resources do not exist individually, but travel in packs or caravans, for both people and organizations, and that some personal resources “emerge from common environmental and developmental conditions and therefore are highly correlated” (Hobfoll et al., 2018, p. 107). The metaphor of resource caravan passageways states that people build and keep their resources within social and environmental conditions that create “resilience or fragility, social skilfulness or social awkwardness, tolerance or intolerance, among the individuals who are exposed to such environments” (Hobfoll et al., 2018, p. 107).

The COR theory has been successfully applied in organizational psychology (Fatima et al., 2018; Halbesleben et al., 2014; Hobfoll et al., 2018) and, consequently, it is an adequate theory for helping us to choose variables that may constitute trait-based leadership resource factors, as well as for interpreting our results. In the following, we explain why we hypothesize that, based on theory and empirical results, the traits we have chosen to examine form trait-based leadership resource factors will be linked with task performance, perceived stress, and work engagement (Bakker & Demerouti, 2008).

### Task Performance, Perceived Stress, and Work Engagement as Keys to an Effective and Successful Leadership

#### Task Performance is Defined by the Leader’s Job Description

Individual work performance is “behaviors or actions that are relevant to the goals of the organization” (Campbell, 1990, p. 704). Individual work performance (Koopmans et al., 2013) consists of three broad dimensions (Koopmans et al., 2011): task performance, contextual performance, and counterproductive work behavior (CWB). In the current study we are focusing on task performance as this has traditionally received the most attention; contextual performance comprises tasks outside the job description, and CWB is not a resource for organizations. Task performance comprises the ability to plan and organize work, the quality of work, the result-orientation, and the ability to work efficiently. An effective leader should possess such abilities to guide the co-workers towards the fulfillment of goals and objectives of an organization. Work performance has been discussed in relation to work engagement and stress (Cropanzano et al., 1997), as well as in relation to emotional intelligence (EI) and Big Five personality traits (McCrae & Costa, 2015). For example, a meta-analysis by O’Boyle Jr. et al. (2011) concluded that EI has incremental validity for predicting work performance, in addition to the Big Five personality traits. This result was valid irrespective of the method used for measuring EI (ability EI or trait EI) and of task performance (self-rated, peer, supervisor, or objective).

#### Low Level of Stress as a Leaders’ Resource to Cope and Lead Effectively

Within the COR theory, Hobfoll (1989) introduced a model of *stress*, which focuses on how people make great efforts to achieve or obtain, retain, and protect their *resources*, in contrast to theories that focus on individual appraisal of stress. According to COR theory, stress occurs when people are experiencing a threat of losing individually valuable key resources. According to Hobfoll (1989) “both perceived and actual loss or lack of gain are envisaged as sufficient for producing stress” (p. 516). To be able to manage such unexpected situations people create new resources. In COR theory, stress is seen as a sequence of stressful events or the appraisal of daily overall pressures, which consequently affect working life. The *desperation principle* (Hobfoll et al., 2018) states that in stressful situations people may become defensive, aggressive, and irrational. The leader is expected lead with a cool head in different situations, try to not become desperate, thus copy well with daily stress, and not to induce stress in co-workers when experiencing own stress. A meta-analysis of Harms et al. (2017) found that ”leadership behaviors were impacted by whether leaders were experiencing stress or not” (p. 185), and it also found an evidence for that leader stress was associated with poorer leadership, making “it difficult for leaders to function effectively in their role” (p. 184). Harms et al. also suggested developing psychological and social resources, “making leaders better capable of operating under stress” (p. 185). Our study focuses on leader’s perception of their daily stress, and relates it to trait-based leadership resources identified by us.

#### Work Engagement as an Effect of Leaders’ Resources

Work engagement is defined as “a positive, fulfilling work-related state of mind that is characterized by vigour, dedication and absorption” (Schaufeli et al., 2002, p. 74). These components of work engagement are individual resources, which the leader brings to the organization. The sum of these individual resources become an important global resource bringing success on the team and organization levels, producing positive outcomes such as superior results (Schaufeli, 2021). A vigorous leader is energetic, active, passionate, and forceful while at work, has the willingness to devote effort to work, and works hard in the face of demanding and unpredictable situations. A dedicated leader feels good, and experiences having great worth, enthusiasm, and inspiration while at work (Kajonius et al., 2016). An absorbed leader gives his or her total attention towards work and may find it difficult to detach from work.

Work engagement is characterized by an intrinsic enthusiasm that drives people to accomplish personal and organizational goals. Highly intrinsically work-motivated leaders are more likely to exert greater effort and to be dedicated and absorbed. Work engagement is positively associated with intrinsic motivation (Schaufeli & Salanova, 2007), with a correlation as high as ~ 0.80 (Malinowska et al., 2018). Engaged people are effective high achievers of work-related demands and are energetic (Schaufeli et al., 2002). People with high levels of work engagement are rated as high-quality performers, both by their co-workers and customers, and are rapidly promoted within the organization (Schaufeli et al., 2002). Engaged people are loyal to their supervisors and show a high level of organizational identification by displaying responsible in-role as well as extra-role behavior at their work (Bakker & Schaufeli, 2008).

#### Which Traits May be Regarded as Important Leadership Resources for Task Performance, Perceived Stress, and Work Engagement?

In our attempt to capture factors of leadership resources which could be helpful for achieving organizational goals, we have identified some valuable human-related traits, such as trait emotional intelligence (EI), empathy, and emotional and spiritual competence forming a compassionate leadership, as well as included traditional personality traits and resources for stress.

#### Trait Emotional Intelligence

Trait EI is a such important emotional resource in working life (e.g., Joseph et al., 2015). People who are emotionally intelligent tend to be able to recognize, control and take care of their own emotions as well as those of other people. Trait EI can be defined as a distinct, stable set of emotionally related self-concepts and adaptive emotional habits (Petrides et al., 2007). Various components of trait EI have been associated with different forms of coping with stress. High trait EI is associated with lower levels of perceived stress (e.g., Petrides & Furnham 2006; Szczygiel et al., 2015). Trait EI is crucial for such working life outcomes as commitment, health, job satisfaction, and job effectiveness (Miao et al., 2017b). Furthermore, leaders high in trait EI may be skillful in communicating with followers on different levels (e.g., e-mail), for instance as regards feedback pertaining to the achievement of goals (Prati et al., 2003); they may have a good control and regulation of own behavior, tolerate frustration, and cope with different changes without intensification of emotional reaction.

Previous research links trait EI positively with work performance (Joseph et al., 2015; O’Boyle et al., 2011; Van Rooy & Viswesvaran, 2004), work satisfaction, organizational commitment, and turnover intentions (Miao et al., 2017a), and leadership effectiveness (Harms & Credé, 2010; Walter et al., 2011). It has also been shown that trait EI is positively linked with personality traits such as extraversion, agreeableness, conscientiousness, and openness (Joseph & Newman, 2010), and that the trait EI-score of leaders is higher than that of their followers (Siegling et al., 2014, 2014). The trait EI levels of leaders affect their followers’ work outcomes (e.g., Miao et al., 2017a). Thus, trait EI seems to be an important trait for including in our analysis of resource factors.

#### Empathy and Compassion

The key ingredient in trait EI is, arguably, a sense of empathy (Skinner & Spurgeon, 2005) and compassion, which can help develop and keep good relations with co-workers and close colleagues (Bakker & Demerouti, 2009), and these should also be investigated as potential valuable human-related components of trait-based resources. Empathy is a feeling that one may have for another person, a capacity to recognize emotions that the other person experiences. Hoffman (2000) described empathy as an affective response that is often associated with the same or similar experience or situation that occurred in one’s life. Empathy has been described as a cognitive process (Deutsch & Madle, 1975), as an emotional process (Håkansson & Montgomery, 2003; Mehrabian & Epsteing, 1972), as an affective feeling that leads to a deeper understanding of another to inspire supportive actions (Zillmann, 2006), and as an emotional state of arousal (Eisenberg et al., 1991). Compassion is defined as an individual response to another person’s suffering (Lilius et al., 2008) closely related to the desire to relieve that suffering (Goetz et al., 2010).

Theoretically (Ronthy, 2006, 2013), an empathetic leader possesses both emotional and spiritual competence (Dåderman et al., 2013), and such a leader should be able to identify the strengths and talents of their followers. These resources are possible to develop in leaders (Ekegren & Dåderman, 2015). Emotional and spiritual competence are, according to Ronthy, important compassionate competence resources for a human-related leadership that not only focuses on the organization’s profit and goals but also on well-being and a morally healthy relationship between the leader and the co-workers. The concept of compassion in the workplace has gained considerable recognition in organizational psychology (Dutton et al., 2014; Shuck et al., 2016), but research on this is still sparse (O’Toole et al., 2020).

## Is being Low on Narcissism a Resource for a Successful Leadership?

Despite a large amount of theoretical literature on narcissism, this construct is still under investigation in organizational psychology (Campbell et al., 2011; O’Reilly & Pfeffer, 2021). Persons with narcissism have an inflated view of themselves and tend to exaggerate their achievements (Campbell et al., 2000). Consistently with this, in top managers narcissism has been found positively correlated with self-rated measures of performance, and negatively correlated with performance as rated by others and as objectively measured (Guedes, 2017). Reports of this effect have received mixed support in the task performance literature (cf. Campbell et al., 2011; Prundeanu et al., 2019), and further research on implications of narcissism for task performance is needed. It seems likely that a combination of favorable traits and the relative absence of unfavorable ones, such as narcissism, may function as a trait-based leadership resource.

There are two main types of narcissism: grandiose and vulnerable. Grandiose narcissism is a key trait characterized by very low empathy, vanity, and high self-love and self-esteem (Johnson et al., 2021; O’Reilly III & Doerr, 2020). In organizations it reflects a belief to be *entitled* to own the right to benefits, items, or activities above the scope of these expected by organizational rules and contracts, a tendency to use different exploitative behaviors to create weaknesses in others and enhance own grandiose sense of self (James et al., 2014). The ruthless prioritization of the leader’s own egoistic needs, irrespective of the detriment caused to others, can lead to adverse effects on interpersonal relationships (Gentile et al., 2012; Keller et al., 2014). At work, narcissists often emerge in positions of authority and power, such as CEOs or political officials, but are prone to impulsive and risky decisions (Nevicka et al., 2011). Past research (e.g., Blair et al., 2017) showed that there are several unethical leadership behaviors in which leaders with narcissism are likely to be engaged. Still, it is commonly thought that some characteristics of narcissism may be helpful in being hired as a leader (Diller et al., 2021).

In addition to entitled behavior, which is typical for the grandiose type of narcissism, vulnerable narcissism is a tendency to hypersensitive and anxious behavior (Wink, 1991), and a tendency to possess deflated self-esteem, depression, anxiety, and a shortage of concern for the need of others. Theoretically and empirically, vulnerable narcissism is not associated with the overt self-reporting (i.e., bragging) of successful organizational behaviors in the past, which contrasts with the grandiose type of narcissism (Freis & Brunell, 2021; Sanecka, 2021). Open demonstration of proactive behaviors in the work context is due to striving for status and power in the organization. There is a positive correlation (0.44) between vulnerable narcissism and perceived stress (Kajonius & Björkman, 2020). It is also positively related to neuroticism (Miller et al., 2011), and emotional exhaustion and negatively to work engagement (Wirtz & Rigotti, 2020). Both types of narcissism are investigated in the current study, and being low on narcissism is considered as a leader resource.

### Performance-Based Self-Esteem as a Part of a Trait-Based Resource

Self-esteem is an often-used variable when COR-theory is applied. It is explicitly defined by this theory as a personal resource for stress reduction (Hobfoll, 1989). The question is whether all types of self-esteem are resources for stress reduction. A low level of contingent, that is, *performance-based self-esteem*, theoretically yields low perceived stress. In contrast, people high in performance-based self-esteem are highly work-engaged, albeit in a toxic way, to heighten their vulnerable self-esteem. Performance-based self-esteem requires constant affirmation by others (Hallsten et al., 2005). It is also unstable; the feeling of self-worth swings quickly depending on task performance and what feedback others provide (Johnson & Forsman, 1995). This may lead to exhaustion and burnout (Blom, 2012; Svedberg et al., 2016). Therefore, similar narcissism, oppositive characteristics of performance-based self-esteem should be a part of a valuable trait-based leadership resource.

#### Personality Traits Conducive for Leadership

Also, personality traits are explicitly defined by the COR theory as personal resources (Hobfoll, 1989), and the personality of leaders has been shown to have important effects on the culture of the organization, shaping the behavior, attitudes, and expectations on others (e.g., Giberson et al., 2009). Personality traits are interlinked with trait EI (Akhtar et al., 2015; Hjalmarsson & Dåderman, 2019). They can be powerful resources in the context of both work motivation (Langelaan et al., 2006) and perceived stress (Ebstrup et al., 2011).

One of the most popular conceptualizations of personality is the Big Five model, which postulates five dimensions: extraversion, neuroticism, agreeableness, conscientiousness, and openness; all known to be strongly related to various forms of motivation (Judge & Ilies, 2002). In addition to the Big Five model, there is the HEXACO model, which is conceptually similar. Emotionality in the HEXACO model differs from neuroticism in Big Five, and HEXACO includes a sixth dimension of *honesty-humility*. This dimension represents “the tendency to be fair and genuine in dealing with others, in the sense of cooperation with others even when one might exploit others without suffering retaliation” (Ashton & Lee, 2007, p. 156). Honesty-humility refers to the degree to which persons exhibit fairness, sincerity, greed avoidance, and modesty – characteristics opposite to narcissism. It may thereby be thought to constitute a leadership resource factor.

Neuroticism, the general tendency to experience negative affect, is a strong predictor of perceived stress. For example, Liu et al. (2021) reported a correlation of 0.48 between neuroticism and perceived stress, which was due to higher levels of perceived threat related to COVID-19 and lower levels of perceived efficacy. According to McCrae & Costa (2015) “people who score high on neuroticism are often prone to having irrational ideas, less able to control their impulses, and less able to cope with stress than others” (p. 10). Thus, scoring low on neuroticism should be a part of one of the leadership resource factors.

Extraversion is associated with a drive for social interaction and stimulation, and in the Liu et al. (2021) study high extraversion contributed to higher levels of perceived stress. But extraversion is also characterized by assertiveness, social activity, and a tendency for being talkative, which should be a part of a coping resource. Rogala et al. (2021) found that extraversion may act as a defense mechanism against perceived stress during exposure to a stress situation such as the COVID-19 pandemic.

Might the complex trait of openness (being unconventional, questioning authority, and open to new ethical, social and political ideas) also be considered as a part of any leadership resource? Its value depends on the requirements of the situation (McCrae & Costa, 2015).

Agreeableness is a tendency to take other people’s perspectives and concerns, and is characterized by a willingness to help others and a believe that others possess similar characteristics. Recently, Blake et al. (2022) argued, and provided evidence with the help of meta-analysis, that nice leaders, high on agreeableness, are effective leaders who build effective relationships with their followers, and inspire, motivate and create well-performing teams. We hypothesize that agreeableness, together with similar valuable human-related traits (empathy, trait EI, compassionate leadership), would be a part of a good-heartedness leadership resource.

Conscientiousness is a rational mastery for controlling impulses (a strong self-control), being punctual, reliable, purposeful and determined in planning, organizing and carrying out tasks. Together with similar traits it should be a part of a leadership resource factor strongly correlated with task performance.

### The Awareness of One’s Accessible Individual Coping Resources for Stress

As emphasized by the COR theory (Hobfoll, 1989), it is important to obtain, retain, and protect different resources. Coping resources are important predictors for the handling of stress (Durm & Glaze, 2002), especially in populations of professionals who perceive stress every day (Dåderman & de Colli, 2014). We here focus on adaptive coping resources, not on maladaptive strategies (such as using or abusing substances), and not on coping strategies. Coping resources are defined by Marting and Hammer (1988) as those resources “inherent in individuals that enable them to handle stressors more effectively, to experience fewer or less intense symptoms upon exposure to a stressor, or to recover faster from exposure” (p. 2), and it is assumed that they play a mediating role in the coping process. There are different coping resources (cognitive, social, emotional, spiritual/philosophical, and physical). Cognitive resources make it possible to maintain a positive view of oneself and of others, and to maintain a generally optimistic attitude. Social resources are provided by the social networks to which a leader belongs, and can provide support in times of stress or when the leader needs support for his/her work engagement. Emotional resources enable a leader to accept and express all kinds of affect, based on the premise that a range of emotional responses aids in relieving the long-term negative consequences of stress. Spiritual/philosophical resources determine the degree to which the actions of a leader are guided by stable and consistent values derived from religious, familial, or cultural traditions, or from personal philosophy. Physical resources reflect the degree to which a leader carries out health-promoting activities believed to contribute to increased physical well-being. It is assumed that physical well-being can decrease the level of negative response to stress and enable faster recovery (Marting & Hammer, 1988). Being conscious about strong coping resources, which were valuable in the past, is assumed to be helpful for limiting the ill effects of stress and recovering faster from a stressful event. Coping is also a strong positive predictor of work engagement (van Loon et al., 2018).

### The Current Study

In this study, we selected relevant traits and resources measured by self-reported scales, examined them by conducting factor analyses and combined them into factors (trait-based leadership resources), and linked these factors to task performance, perceived stress, and work engagement, three crucial ingredients in successful leadership.

### Aim of the Study

The aim was to test the hypothesis, derived from Hobfoll’s motivational Conservation of Resources (COR) theory, that there are trait-based leadership resource factors, which are differentially correlated to the leaders’ task performance, perceived stress, and work engagement.

## Materials and Methods

### Participants

The participants were 344 leaders (58% women) working in Sweden. Their mean age was 49 years (*SD* = 8.6, range 23–65). The average duration of experience as a leader in the current managerial position was five years (*SD* = 6.3, range ~ 1–38); 19.4% worked at a superior level, 68.6% at an intermediate level, and 12% at a lower level (e.g., as a group leader). The leaders worked in such fields as industrial production, social services, nursing and care services, and education; 26% worked for two private-owned companies, 41% for four municipalities in western Sweden, 19% for a private-owned organization in a municipality close to Stockholm, and 13% worked for two state organizations. In total, data of leaders from nine organizations were sampled; 254 leaders were at managerial positions at human-oriented organizations, while 87 of them worked in such positions at manufacturing industries.

### Procedure and Sampling Strategy

Since 2002, University West has the Swedish Government’s commission to develop a model called AIL [Work Integrated Learning], which is University West’s academic profile. The current data were sampled within a project, entitled *Det medmänskliga ledarskapet* [The human-related leadership]. In short, in this project we examine the concept of *human-related leadership* which, according to theory of Ronthy (2013), consists of three parts. The rational part is related to concrete tasks related to the managerial assignment, such as task performance. The emotional part is about self-knowledge and how the person in a managerial position handles their and others’ feelings, as well as the degree of empathy and the degree to which they can manage relationships with others. The spiritual part is related to the meaning of the managerial assignment and is about vision, values, and being able to see context through a holistic approach. The leadership role is not formal, and it is earned depending on how good the manager is at leading himself and his employees. A person in a managerial position should be able to balance and be both task-oriented and human-oriented.

The first author contacted Human Resources (HR) managers from six municipalities and organizations in the Western Swedish region to invite leaders from their organizations to participate in the study. The HR managers were provided with the project description. They presented the project to leaders working at a superior level. The HR managers from organizations that agreed to participate provided mailing lists with the potential participants. Also, several private organizations in the same region and in Stockholm were contacted. Via email, the first, third and fourth authors sent out an invitation to leaders in these organizations, along with the project description and a project ethics statement. These authors also administered the database. The leaders provided their responses via a web questionnaire provided by the free Internet Google Form software. Due to the survey’s anonymous design, entailing that the researchers could not know whether a leader had already responded, all leaders on the mailing lists received three reminder mails. All data were sampled during a period of five weeks in the autumn of 2017. The response rates from the organizations were satisfying; 73% (range 65–81%).

### Test Instruments

The criteria for selecting test instruments included high face validity for leaders and conceptual appropriateness (e.g., being a potential trait-based leadership resource). Other criteria were free use and established psychometric properties. Swedish versions of the test instruments were used. All the employed test instruments measure traits, perceptions, and dispositions, measured by self-reports. A high scale score indicates a high value of the measured variable. Details regarding items, descriptive statistics of the scales, including Cronbach’s alpha reliabilities and mean inter-item correlations in the present group, are presented in Table 1. In the following, we present key references to these measures, as well as challenges experienced with some items while assessed among leaders.

#### Variables Assumed being Important for an Effective and Successful Leader

Task performance was measured by the **Individual Work Performance Questionnaire (IWPQ)**. The IWPQ (Koopmans et al., 2013) measures individual work performance using responses ranging from *Seldom* to *Always*. The IWPQ comprises three scales: Task Performance, Contextual Performance, and Counterproductive Work Behavior (CWB). The Swedish version is available (Dåderman et al., 2020, Supplementary material). The authors have permission to translate, adapt, validate, and use the IWPQ from the copyright holders. In the current study, only the Task Performance Scale, consisting of five items, was analyzed.

Perceived stress was measured by ten items from the **Perceived Stress Scale** (PSS-10). PSS-10 (Cohen & Williamson, 1988) measures the degree to which different daily situations are considered as stressful, using responses ranging from *Never* to *Very often*. PSS-10 is often used as an estimate of perceived stress. The Swedish version is available (Nordin & Nordin, 2013, Table 4).

Work engagement was measured by the **Utrecht Work Engagement Scale** (UWES-9) (Schaufeli & Bakker, 2004), which measures the global level of work engagement by nine items reflecting vigor, dedication, and absorption, using responses ranging from *Never* to *Always/every day*. The Swedish version is available (the UWES Manual, p. 52) (UWES (wilmarschaufeli.nl).

#### Scales Forming Trait-Based Leadership Resource Factors

##### Trait Emotional Intelligence Questionnaire-Short Form (TEIQue-SF).

TEIQue-SF is a short form of the TEIQue (Petrides, 2009) using responses ranging from *Completely disagree* to *Completely agree*. The TEIQue-SF comprises 30 items selected from the 15 facets of the TEIQue’s four scales: Emotionality, Self-control, Well-being, and Sociability. The items were selected based on their correlations with the corresponding total facet scores. The TEIQue-SF is especially designed to be used as a global measure of trait emotional intelligence (trait EI). The Swedish version of the TEIQue-SF has recently been validated in relation to work performance and personality traits by Hjalmarsson and Dåderman (2022), and psychometrically evaluated in a sample of working people (*N* = 973) by Dåderman and Kajonius (2022). It is available: https://psychometriclab.com/translations-of-teique/).

##### Leadership Intelligence Questionnaire (LIQ), Version 3 (LIQ3).

LIQ3 is a short and revised form of the LIQ (Dåderman et al., 2013), used to measure leadership competence (Ronthy, 2006, 2013), assumed to be a set of emotional, social, and practical competences and/or skills and work resources, using responses ranging from *Strongly disagree* to *Fully agree*. The LIQ3 comprises three scales: Emotional Intelligence (EQ), Spiritual Intelligence (SQ), and Rational Intelligence (RQ). The LIQ3 has been validated (Hallberg & Skog, 2017). The authors have permission from the copyright holders to use, adapt and validate the LIQ3.

In the current study, the mean scores of the EQ and SQ scales were highly correlated (0.53), and in a preliminary factor analysis they formed the same trait-based leadership factor. To keep the number of variables as low as possible, we combined EQ and SQ into a new variable, named *compassionate leadership competence* (EQ + SQ)/2.

##### Coping Resources Inventory (CRI).

The CRI (Marting & Hammer, 1988; translated into Swedish by Ekecrantz & Norman, 1991) identifies a person’s accessible resources for managing stress. The CRI was constructed to facilitate an emphasis on resources rather than deficits. It measures five forms of coping resources: cognitive, social, emotional, spiritual/philosophical, and physical. For example, social resources are provided by the social networks to which a person belongs, and they can provide support in times of stress. The CRI measures such resources as precursors of coping behavior, and *not* as coping strategies (i.e., responses to a stressor or to prolonged stress). The CRI uses responses ranging from *Never or rarely* to *Almost always or always*. All items have a recall period of six months. The authors have permission from the copyright holders to use this instrument.

##### Interpersonal Reactivity Index (IRI).

The IRI (Davis, 1980, Table 3; Davis, 1983) measures empathy using responses ranging from *Does not describe me well* to *Describes me very well*. Cliffordson (2002) validated the Swedish version of the IRI scale (translated by Kulich and Bengtsson). The IRI comprises four scales. Two of them measure adaptive forms of empathy; cognitive empathy (Perspective Taking) and affective empathy (Empathic Concern), while remaining two measure maladaptive forms of empathy; cognitive empathy (Fantasy) and affective empathy (Emotional Distress). Only items from the Empathic Concern and Perspective-Taking scales were sampled.

##### Hypersensitive Narcissism Scale (HSNS).

The HSNS (Hendin & Cheek, 1997) measures vulnerable narcissism using responses ranging from *Very uncharacteristic or untrue, strongly disagree* to *Very characteristic or true, strongly agree*. The Swedish version (translated by Björkman and Kajonius, revised by Hellström) was used. The HSNS is a valid and well-established scale measuring vulnerable narcissism (Kajonius & Björkman, 2020).

##### Short Dark Triad (SD3).

The SD3 (Jones & Paulhus, 2014) comprises three scales, but only items from the subclinical Narcissism (grandiose type) scale were sampled. The SD3 uses responses ranging from *Strongly disagree* to *Strongly agree*. The Narcissism Scale had four items that did not fit well in this sample, and Cronbach’s alpha became higher when these items were deleted: *People see me as a natural leader*, *I hate being the center of attention* (reversed), *I feel embarrassed if someone compliments me* (reversed), and *I am an average person* (reversed). The Swedish version (translated and adapted by Lindén and Dåderman) is published (Dåderman & Ragnestål-Impola, 2019, Supplementary material).

##### Mini International Personality Item Pool-6 Inventory (Mini-IPIP6).

The Mini-IPIP6 (Donnellan et al., 2006; Goldberg, 1999; Sibley, 2012) measures common personality traits comprising six scales: Neuroticism, Extraversion, Openness to Experience, Agreeableness, Conscientiousness, and Honesty-Humility. The Mini-IPIP6 uses responses ranging from *Strongly disagree* to *Strongly agree*. The Openness to Experience (being imaginative, independent-minded, and autonomous) scale had lower Cronbach’s alpha, which, when measured with a few items only, is in line with past research (Woo et al., 2014). The Openness scale had, however, a sufficient mean inter-item correlation. The low value of Cronbach’s alpha found in this case is not surprising, because this scale comprises only four items. We deleted the following item from the Openness Scale: *I have a vivid imagination*. This deletion improved the scale’s Cronbach’s alpha from 0.57 to 0.61. The Swedish version (translated and adapted by Backström, Dåderman, Grankvist, Kajonius, and Lundin) is available (Dåderman & Ragnestål-Impola, 2019, Supplementary material).

##### Performance-Based Self-Esteem (PBSE).

The PBSE scale (Hallsten et al., 2005) measures contingent self-esteem using responses ranging from *Fully disagree* to *Fully agree*. The context-free version was used. The Swedish version is published (Hallsten et al., 2005, Appendix p. 39).

### Control Variables

In addition, we collected **background information** and some of these variables were used as control variables (sex, age, and number of years as a leader in the current managerial position). We also used the **Balanced Inventory of Desirable Responding (BIDR-6)** (Bobbio & Manganelli, 2011, originally created by Paulhus, 1984, 1991). BIDR-6 comprises two measures for socially desirable responding. These can be separated into *unconscious* self-deceptive enhancement and *conscious* impression management (Parmač Kovačíć, 2021; Paulhus, 2002). Self-deceptive enhancement is a stable personality characteristic, while impression management depends on the characteristics of the situation a person is in (Paulhus, 2002). The permission to translate and adapt to Swedish, as well as use it was given by Paulhus to the first author; it was translated by Grankvist and Lundin. Swedish version of the BIDR-6 has good psychometric properties. The validation study (Dåderman et al., unpublished) comprises data sampled between 2017 and 2019 in both male and female participants from different settings; nurses (*N* = 939), inmates (*N* = 287), working people from different organizations (*N* = 333), and managers (*N* = 344). In the current sample, CFA indices indicated adequate fit: Chi-square = 218.15, *df* = 103, *p* < .001; RMSEA = 0.057 [0.047, 0.068], *p* of close fit (PCLOSE) = 0.13.

#### Ethical Statement.

This study was carried out in accordance with the general recommendations of the Swedish Research Council. Data were sampled during 2017, and according to Swedish law (2003:460, §2), separate ethical approval was not required when a study was performed as part of a B.Sc. thesis, and/or when data were gathered anonymously. Both conditions were met. No link between participants and personal data may be identified. The participants were informed of the study through the initial formal notice prior to accessing the web-based questionnaire. As per the Swedish Research Council’s guidelines, the formal notice listed the four research principles, namely the information requirement, the consent requirement, the confidentiality requirement, and the utility requirement. The participants were informed that their participation in the study was voluntary and that their responses would be treated in confidence. The participants were guaranteed that their answers were anonymous; that they could not be identified by the Internet Protocol (IP) address of their computer of phone, and that they could cancel their participation in the questionnaire at any time. No questions were asked regarding education, marital status, socioeconomic status, or ethnicity. All questions and statements were non-mandatory. All the participants gave their written informed consent in accordance with the Declaration of Helsinki. Each participating organization’s CEO and HR-director approved this study and provided the mailing’s list of potential leaders. The data sampling and data management in this project has been approved by Fyrbodal Municipalities [Kommunakademin Väst] and University West (Diary no. 100127).

## Results

### Descriptive Statistics

All statistical analyses were performed in SPSS 25. In preparing for an exploratory factor analysis of the study scales (19 variables), all single missing values (< 1%) were replaced by the mean for all cases. Internal consistency of the scales was determined using Cronbach’s alpha (Cronbach, 1951). Because we used short scales comprising a few items, we also calculated mean inter-item correlations. Descriptive statistics of the scales used scales are presented in Table 1 for all 344 cases. The table with intercorrelations between variables used in in the factor analyses (see below) is presented in the Supplementary Material (Table S1).

**Table 1:** *Descriptive statistics for the used scales*

Variable	Example of item	No. items	Scale range	Min	Max	Mean	*SD*	*S*	*K*	*α*	*M*_*icc*_
Core elements of an effective and successful leadership											
Task performance (IWPQ)	During the past three months…										
	I was able to plan my work so that I finished it on time	5	0–4	0.60	4.00	2.68	0.57	-0.34	0.45	0.72	0.35
Perceived stress (PSS10)	In the last month… How often have you felt stressed?	10	0–28	1.00	27.00	12.82	4.52	0.13	-0.02	0.79	0.28
Work engagement (UWES-9)	I am enthusiastic about my job	9	0–6	1.33	6.00	4.48	0.77	-0.79	1.22	0.89	0.48
Traits and resources *Trait emotional intelligence*											
Global trait EI (TEIQue-SF)	Expressing my emotions with words is not a problem for me	30	1–7	4.00	6.73	5.80	0.42	-0.55	0.96	0.80	0.13
*Leadership competence (LIQ3)*											
Emotional competence	I inspire others to be creative	7	1–7	2.29	6.71	5.42	0.61	-0.66	1.42	0.67	0.25
Spiritual competence	I act in accordance with my values	7	1–7	2.86	7.00	5.89	0.57	-0.89	2.22	0.67	0.22
Rational competence	I specify strategies to achieve the goals set	7	1–7	2.43	7.00	4.93	0.75	-0.30	0.36	0.70	0.27
*Coping resources (CRI)*	During past six months…										
Cognitive	I actively look for the positive side of people and situations	9	9–36	17	36	28.86	3.57	-0.39	0.11	0.78	0.30
Social	I am part of a group, other than my family, that cares about me	13	13–52	24	48	39.18	4.17	-0.19	-0.07	0.80	0.24
Emotional	I can show it when I am sad	16	16–64	26	63	45.48	6.32	0.003	-0.45	0.84	0.24
Spiritual/Philosophical	I accept problems that I cannot change	11	11–44	20	43	27.45	4.12	1.01	1.41	0.69	0.17
Physical	I have plenty of energy	11	11–44	15	44	29.47	5.40	0.03	-0.39	0.82	0.30
*Empathy (IRI)*											
Empathic concern	I often have tender, concerned feelings for people less fortunate than me	7	0–28	9.00	28.00	21.79	3.48	-0.51	0.44	0.69	0.26
Perspective taking	I sometimes try to understand my friends better by imagining how things look from their perspective	7	0–28	6.00	28.00	21.15	3.58	-0.63	1.12	0.76	0.32
*Narcissism*											
Vulnerable (HSNS)	My feelings are easily hurt by ridicule or the slighting remarks of others	10	10–50	10	38	20.97	5.12	0.43	0.12	0.73	0.23
Grandiose (SD3)	People see me as a natural leader	5	1–5	1.00	5.00	2.33	0.67	0.45	0.41	0.62	0.26
*Personality traits (Mini-IPIP6)*											
Neuroticism	I have frequent mood swings	4	1–7	1.00	6.75	2.72	1.03	0.65	0.67	0.69	0.37
Extraversion	I am the life of the party	4	1–7	1.50	7.00	4.53	1.17	-0.21	-0.40	0.70	0.37
Openness to experience	I have a vivid imagination	3	1–7	2.67	7.00	5.84	1.04	-0.76	-0.34	0.61	0.35
Agreeableness	I sympathize with others’ feelings	4	1–7	3.00	7.00	5.76	0.79	-0.60	-0.04	0.64	0.31
Conscientiousness	I like order	4	1–7	1.75	7.00	5.55	1.04	-0.81	0.28	0.73	0.40
Honesty-Humility	I deserve more things in life (R)	4	1–7	2.00	7.00	5.52	1.08	-0.58	-0.35	0.64	0.31
*Self-esteem (PBSE)*											
Performance-based self-esteem	I think that I sometimes try to prove my worth by being competent	4	1–5	1.00	5.00	2.89	0.95	-0.04	-0.72	0.81	0.51
*Social desirability (BIDR6)*											
Self-deceptive enhancement	I am very confident in my judgments	8	1–7	2.13	6.50	4.52	0.75	-0.06	-0.03	0.70	0.23
Impression management	There have been occasions when I have taken advantage of someone	8	1–7	1.88	7.00	5.04	0.89	-0.32	-0.16	0.67	0.20

Notes: See the *Materials and Methods* for the abbreviations and a description of the used instruments. *S* = skewness. *K* = kurtosis. *α* = Cronbach’s alpha. *M*_*icc*_ = mean inter-item correlation. R = reversed. In the current study, two scales (Emotional competence and Spiritual competence) from the LIQ3 were strongly correlated (0.53) and have been combined to one measure of *Compassionate leadership competence* by an averaging the scales’ means

### The Trait-Based Leadership Resource Factors

We have applied a series of procedural remedies to prevent eventual common method variance (CMV) bias, and also run the Harman’s single factor test to ensure that the extracted factors are free from CMV (see Rodriguez-Ardura & Meseguer-Artola, 2020). There was no problem with CMV in the current data since the total variance extracted by one factor was only 11.8%.

#### Exploratory Factor Analysis

We complied with Nunnally’s (1978) recommendation that one should have ten times as many participants as variables. We also satisfied the *Rule of 200* (Gorsuch, 1983), as well as Tabachnick and Fidell’s (2019) suggestion that it is prudent to have at least 300 cases. The Kaiser-Meyer-Olkin measure of adequacy (KMO) (Kaiser, 1974) was 0.84, *p* < .001 (meritorious, according to Hutcheson & Sofroniou, 1999). A preliminary exploratory factor analysis (by principal axis factoring, PAF) was conducted on raw scores of all variables. We have chosen PAF because this technique is the most commonly used, understood, and recommended factor extraction technique (Tabachnick & Fidell, 2019). Five factors had eigenvalues satisfying Kaiser’s criterion of an eigenvalue above 1, and in combination explained 62.9% of the variance, but the five factors did not show a theoretical meaningful relevance for purpose for this study. In this solution, among others, one of the complex variables (Dåderman & Kajonius, 2022), measuring the global trait of emotional intelligence, loaded on more than one factor. To decide how many factors to retain, we conducted a parallel analysis (Hayton et al., 2004), which indicated that four factors should be retained. This was supported by inspection of the scree plot. An oblique rotation showed that two factors correlated as highly as 0.41 (approximately 10% overlapping variance). Therefore, oblimin rotation was used. Table 2 shows the factor loadings after rotation. We identified four trait-based leadership resource factors, which in combination explained 57.4% of the variance. We changed signs of loadings of variables on Factor 2_rev_ (renamed as *Modesty*). This was done in order to describe this factor as a resource.

**Table 2:** *Summary of exploratory factor analysis*

Variable	Factor 1 Efficient Coping	Factor 2_rev_ Modesty	Factor 3 Good-Heartedness	Factor 4 Rational Mastery
Cognitive coping resource (CRI)	0.85			
Emotional coping resource (CRI)	0.80			
Social coping resource (CRI)	0.77			
Global trait EI (TEIQue-SF)	0.51			
Spiritual/Philosophical (CRI)	0.47			
Extraversion (IPIP6)	0.43			
Physical coping resource (CRI)	0.35			
Openness (IPIP6)	0.31			
Grandiose narcissism (SD3)	0.32	− 0.74		
Honesty-Humility (IPIP6)		0.61		
Performance-based self-esteem (PBSE)		− 0.52		
Vulnerable narcissism (HSNS)		− 0.50		
Neuroticism (IPIP6)		− 0.40		
Agreeableness (IPIP6)			0.73	
Empathic concern (IRI)			0.62	
Perspective taking (IRI)			0.52	
Compassionate leadership competence (LIQ3)			0.50	
Rational competence (LIQ3)				0.74
Conscientiousness (IPIP6)				0.60
Eigenvalues	5.51	2.10	1.88	1.37
% of variance	29.02	11.04	9.89	7.20

Notes: _rev =_ Loading signs are reversed to reflect a resource. Principal axis factoring and oblimin rotation with Kaiser normalization. Variables are ordered and grouped by size of factor loadings to facilitate interpretation. Only factor loadings at least 0.32, except for openness which showed a relatively low factor loading, are reported and interpreted. See the *Materials and Methods* for the abbreviations and Table 1 for a brief description of the variables

For illustrative purposes, we ran a simple CFA on the 19 scales used in the EFA. The figure is presented in the Supplementary Material (Figure S1).

### What Constitutes Trait-Based Leadership Resource Factors?

We interpreted the four extracted factors as representing broad leadership trait-based resources and named them accordingly. Factor 1 (Efficient Coping) represents traits such as adaptive coping resources for stress and emotional intelligence, but also healthy externalizing traits such as energy (extraversion) and intellectual creativity (i.e., openness). The loadings of Factor 2 were reversed to enhance its interpretability as a resource. Factor 2_rev_ (Modesty*)* represents trait honesty-humility (fairness, sincerity, greed avoidance, and modesty) combined with the *relative absence* of (negative loadings for) the traits narcissism (both vulnerable and grandiose), performance-based self-esteem, and neuroticism. This combination of traits reflects good cooperation and fairness. Factor 3 (Good-Heartedness) represents morally and socially good traits and abilities such as agreeableness, compassionate leadership competence (emotional and spiritual), and empathy (empathic concern and perspective taking). Factor 4 (Rational Mastery) represents traits such as striving after high task performance, rational responsibility (i.e., leadership rational competence), and conscientiousness. The four factors are analyzed and discussed in the Discussion section, where we also provide our suggestions as to what they might mean for the trait-based leadership.

### Are the Four Trait-Based Leadership Factors *Differentially *Correlated to the Leaders’ Task Performance, Perceived Stress, and Work Engagement?

#### Correlation Analyses

Factor scores were calculated by the regression method. We then computed correlations of the factor scores from the four extracted broad trait-related leadership resource factors with task performance, perceived stress, and work engagement. We also computed the corresponding partial correlations, controlled for age, sex, number of years in the current managerial position, and the two measures of socially desirable responding. For illustrative purposes, Table 3 presents simple correlations along with partial correlations. The *p* values were Bonferroni adjusted (see the note to Table 3) separately for the 32 simple correlations relevant for the current study and for the 12 partial correlations.

**Table 3:**
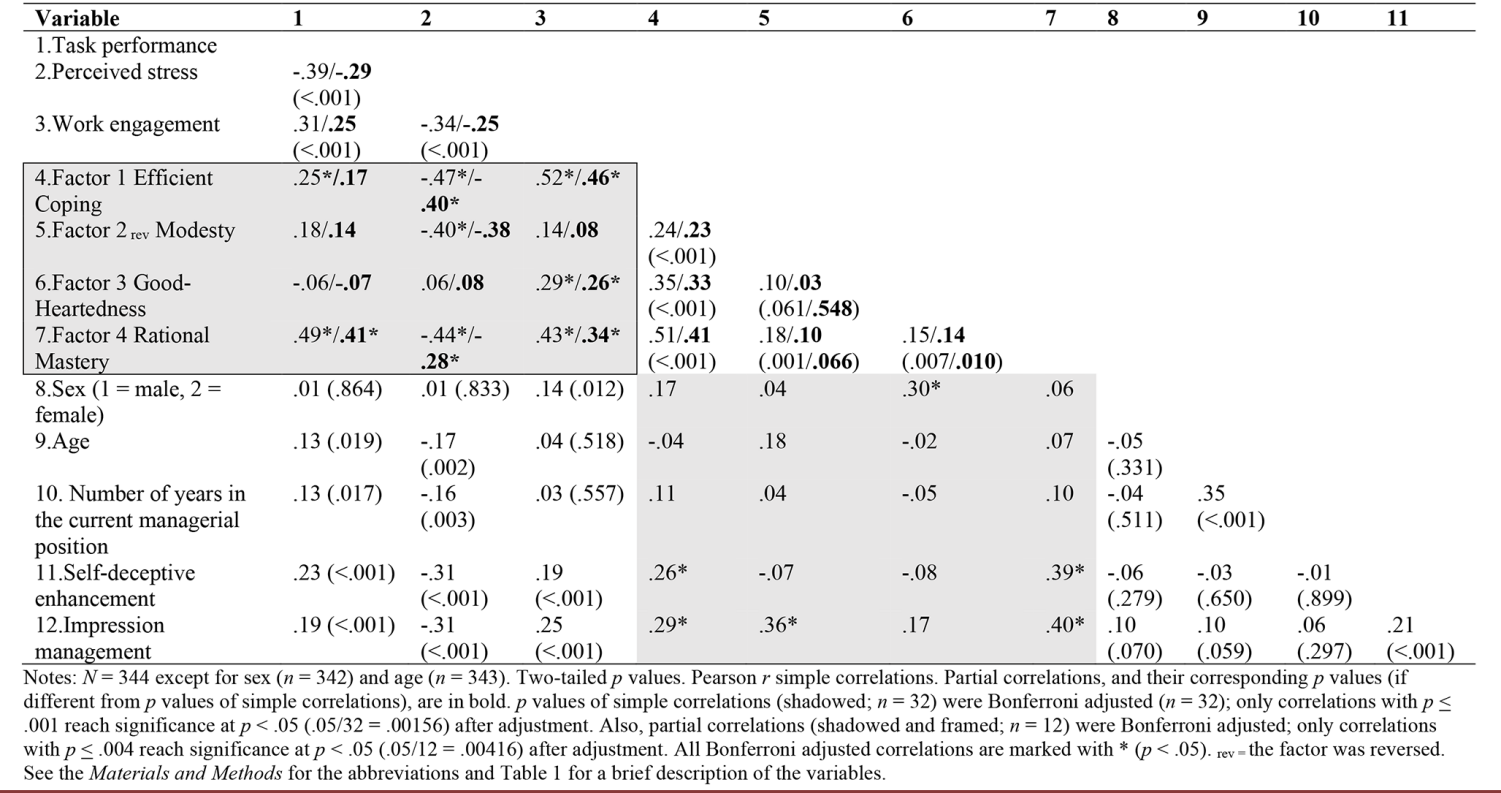
Correlations between the studied variables

Table 3 shows that the four trait-based leadership factors were *differentially* correlated to the leaders’ task performance, perceived stress, and work engagement, which was in line with our hypothesis. With Bonferroni adjusted partial correlations, only Rational Mastery was significantly positively correlated with task performance. Efficient Coping, Modesty, Rational Mastery were negatively correlated with perceived stress, and all factors except Modesty, but including the fourth (Good-Heartedness) were positively correlated with work engagement. The partial correlation with task performance was large for Rational Mastery, almost medium for Efficient Coping, and small for Modesty^1^. We found almost zero correlation between Good-Heartedness and task performance. All partial correlations of the three factors that were negatively and significantly correlated to perceived stress were considered as large (for Rational Mastery it was almost large). The correlation with work engagement was large for Efficient Coping as well as Rational Mastery, while it was medium but almost large for Good-Heartedness (see Table 3).

#### Additional Analyses

To help characterize the four leadership resource factors, we also examined whether the extracted factor scores were different between men and women. To estimate the mean differences, we used the independent *t*-test. To estimate the effect sizes (Cohen’s *d*), we used an online calculator [Free Effect Size (Cohen’s d) Calculator for a Student t-Test - Free Statistics Calculators (danielsoper.com)].

On average, females (*n* = 199) had significantly higher mean factor scores (*M* = 0.14, *SD* = 0.95) than males (*n* = 143; *M* = -0.20, *SD* = 0.92) on Factor 1 Efficient Coping; this difference, -0.33, 95% CI [-0.53, -0.13], was significant, *t*(340) = 3.25, *p* = .001, *d* = 0.51. Further, females had significantly higher mean factor scores (*M* = 0.23, *SD* = 0.81) than males (*M* = -0.32, *SD* = 0.91) on Factor 3 Good-Heartedness; this difference, -0.54, 95% CI [-0.73, -0.36], was significant, *t*(340) = 5.82, *p* < .001, *d* = 0.63. There were no significant sex differences regarding Factor 2_rev_ Modesty (*d* = 0.07) nor Factor 4 Rational Mastery (*d* = 0.11). These results are in line with the correlation analyses presented in Table 3. (With Bonferroni adjustment, see above, only correlations between sex and Efficient Coping, and Good-Heartedness were significant at *p* < .05.)

To estimate correlations between the four factors and the remaining background variables (age and number of years in the current managerial position), we used the Pearson correlation coefficient. Also, these results were Bonferroni adjusted. Only the factor expressing modesty (Factor 2_rev_) was significantly (*p* < .05) related to age (see Table 3).

It should be noted that we included checks for socially desirable responding. Impression management (i.e., consciously lying or facing) was positively correlated with all factors, while self-deceptive enhancement (a form of unconscious positive bias in item responses) was positively correlated with only Efficient Coping and Rational Mastery, showing almost zero correlations with Modesty and Good-Heartedness. Additionally, we examined sex differences in the mean scale scores for the two measures for socially desirable responding. There were no significant sex differences in the mean scale scores for the *unconscious* self-deceptive enhancement (*d* = 0.12) nor *conscious* impression management (*d* = 0.20).

Given the complexity of resources we also conducted regression analyses with task performance, perceived stress, and work engagement as dependent variables and factor scores of the four leadership resource factors, controlled for sex, age and social desirability, as the independent variables. These results are presented in Table 4.

**Table 4:** *Linear models of predictors of task performance, perceived stress and work engagement (N = 344) with 95% bias corrected and accelerated confidence intervals reported in parentheses*

Variable	B	*SE* B	β	*p*
***Task performance*** *R*^*2*^ = 0.10 for Step 1 (*p* < .001); Ϫ*R*^*2*^ = 0.18 for Step 2 (*p* < .001)				
Step 1				
Constant	1.09 (0.456, 1.709)	0.31		< 0.001
Sex (1 = male, 2 = female)	0.22 (-0.100, 0.147)	0.06	0.02	0.715
Age	0.01 (0.001, 0.014)	0.003	0.12	0.024
Self-deceptive enhancement	0.17 (0.088, 0.248)	0.04	0.22	< 0.001
Impression management	0.08 (0.010, 0.157	0.04	0.13	0.016
Step 2				
Constant	2.25 (1.614, 2.877)	0.32		< 0.001
Sex	0.04 (-0.085, 0.154)	0.06	0.03	0.528
Age	0.006 (0.000, 0.012)	0.003	0.09	0.074
Self-deceptive enhancement	0.05 (-0.032, 0.121)	0.04	0.06	0.256
Impression management	-0.02 (-0.090, 0.052)	0.04	− 0.04	0.520
Efficient Coping	0.02 (-0.046, 0.091)	0.03	0.04	0.515
Modesty	0.06 (-0.001, 0.123)	0.03	0.10	0.064
Good-Heartedness	-0.09 (-0.154, -0.029)	0.03	− 0.15	0.006
Rational Mastery	0.30 (0.226, 0.379)	0.04	0.46	< 0.001
***Perceived stress*** *R*^*2*^ = 0.18 for Step 1 (*p* < .011); Ϫ*R*^*2*^ = 0.26 for Step 2 (*p* < .001)				
Step 1				
Constant	29.85 (25.427, 34.181)	2.16		< 0.001
Sex	0.08 (-0.814, 0.931)	0.45	0.01	0.859
Age	-0.08 (-0.127, -0.023)	0.03	− 0.14	< 0.001
Self-deceptive enhancement	-1.63 (-2.194, -1.067)	0.30	− 0.27	< 0.001
Impression management	-1.21 (-1.726, -0.696)	0.26	− 0.24	< 0.001
Step 2				
Constant	20.95 (16.895, 24.889)	2.03		< 0.001
Sex	0.11 (-0.703, 0.882)	0.41	0.01	0.784
Age	-0.06 (-0.102, -0.012)	0.02	− 0.11	0.009
Self-deceptive enhancement	-0.97 (-1.520, -0.427)	0.28	− 0.16	< 0.001
Impression management	-0.21 (-0.660, 0.270)	0.24	− 0.04	0.386
Efficient Coping	-1.67 (-2.169, -1.182)	0.26	− 0.35	< 0.001
Modesty	-1.49 (-1.947, -1.037)	0.23	− 0.29	< 0.001
Good-Heartedness	1.10 (0.636, 1.568)	0.24	0.22	< 0.001
Rational Mastery	-0.78 (-1.287, -0.249)	0.27	− 0.15	0.006
***Work engagement*** *R*^*2*^ = 0.10 for Step 1 (*p* < .001); Ϫ*R*^*2*^ = 0.23 for Step 2 (*p* < .001)				
Step 1				
Constant	2.44 (1.573, 3.260)	0.43		< 0.001
Sex (1 = male, 2 = female)	0.20 (0.039, 0.379)	0.08	0.13	0.014
Age	0.002 (-0.007, 0.012)	0.005	0.02	0.644
Self-deceptive enhancement	0.17 (0.060, 0.274)	0.05	0.16	0.003
Impression management	0.17 (0.083, 0.260)	0.05	0.20	< 0.001
Step 2				
Constant	3.97 (3.155, 4.748)	0.41		< 0.001
Sex	0.04 (-0.107, 0.200)	0.08	0.03	0.587
Age	0.004 (-0.005, 0.012)	0.004	0.04	0.395
Self-deceptive enhancement	0.03 (-0.085, 0.136)	0.06	0.03	0.631
Impression management	0.03 (-0.051, 0.111)	0.04	0.04	0.489
Efficient Coping	0.29 (0.187, 0.387)	0.05	0.35	< 0.001
Modesty	-0.01 (-0.099, 0.080)	0.05	− 0.02	0.762
Good-Heartedness	0.11 (0.012, 0.211)	0.05	0.13	0.010
Rational Mastery	0.19 (0.064, 0.293)	0.06	0.21	< 0.001

Notes: *B* = unstandardized coefficient; *R*^*2*^ = coefficient of determination; Ϫ*R*^*2*^ = coefficient of determination change. Confidence intervals and standard errors based on 5,000 bootstrap samples

Table 4 shows that the four factors of managerial resources, with all control variables, explained 28%, 33%, and 44%, respectively, of the variability in task performance, perceived stress, and work engagement. In summary, our results support the hypothesis, derived from Hobfoll’s motivational Conservation of Resources (COR) theory, that there are trait-based leadership resource factors, which are differentially correlated to the leaders’ task performance, perceived stress, and work engagement.

## Discussion

To our knowledge, this is one of the first studies to propose, based on relevant traits and resources, broad trait-based leadership resource factors. Our four factors are Efficient Coping, Modesty, Good-Heartedness, and Rational Mastery. In this study, we were able to show that factor scores of these leadership resource factors are differently correlated with task performance, perceived stress, and work engagement. Our results are in line with the COR theory, a motivational theory that takes stress into account. We found that individual traits combine into valuable leadership resources for increased work engagement and decreased perceived stress. Our results add to the knowledge of this by examining trait resources through the lens of the COR theory, in a group of leaders in Swedish private and public organizations. Let us elaborate somewhat on the four proposed trait-based leadership resource factors.

### Efficient Coping

The first resource factor, Efficient Coping (see Table 2 and Figure S1) comprises positive loadings of coping resources for stress (cognitive, social, emotional, spiritual/philosophical, and physical), the global trait EI (i.e., the emotionality, self-control, well-being, and sociability dimensions of trait EI). It also comprises extraversion and openness.

What do all these positive factor loadings mean for the trait-based leadership resource factor of Efficient Coping? Possessing high factor scores of Efficient Coping is determined by characteristics of the captured traits. Three of the coping resources for stress; social (belonging to strong social networks), emotional (enabling to accept and express all kinds of affects), and cognitive (maintaining a positive view of oneself) have their largest correlations with this factor (see Table 2 and Figure S1). Physical coping has the lowest correlation with this factor, indicating that this type of coping does not influence Efficient Coping so much than other types of coping resources. Also, global trait EI loaded on this factor, and thus, this factor has very similar characteristics as global trait EI, indicating a high level of self-esteem and awareness of enjoyment and pleasure together with positive thinking, good regulation and control of own emotional responses, and coping with stress, empathy, communicating feelings, being aware of the perspectives of other people in a situation, having a high social competence, as well as an ability to show strong social skills and to be assertive and influence others. The interpersonal components of this factor, such as extraversion and openness, are theoretically assumed to be associated with a greater tendency to use coping strategies that involve seeking support from others, which, according to societal standards, encourages women to be more interpersonal than men. Research shows that females have higher mean scores than men on all Big Five personality traits, including extraversion (Mac Giolla & Kajonius, 2019). It is therefore not surprising that female leaders had significantly higher mean Efficient Coping factor scores than male leaders; this difference was large (Gignac & Szodorai, 2016).

With perceived stress Efficient Coping, controlled for background factors and socially desirable responding, had a strong negative correlation (see Table 3), and the highest beta coefficient in the corresponding regression (see Table 4). With task performance it had a typical (medium) correlation. But in the regression analysis, which helps to control the shared variance between resources, this association vanished (see Table 4).

The Efficient Coping factor comprises all five forms of coping resources for stress (Marting & Hammer, 1988). The role of such resources for stress management is well-known. For instance, emotion-focused coping has shown negative associations with distress and worry (Matthews et al., 2000). Extraversion is a part of this leader resource, and past research showed negative association of extraversion with stress (e.g., Deary et al., 1996; Vollrath, 2000).

Coping is a strong positive predictor of work engagement (van Loon et al., 2018), which was also evident in our study. Theoretically, cognitive resources enable leaders to maintain a positive view of themselves (i.e., self-concept) and of others. The role of a positive self-concept in the adaptation to stress is well-known (Pearlin & Schooler, 1978). Self-concept clarity is positively related to work engagement and work motivation (Balundė & Paradnikė, 2016). Such resources may serve to give a meaning to potentially stressful events and to prescribe strategies for responding effectively. In previous research, both emotional and spiritual coping have been found to be negatively related to perceived fear (Zeidner & Hammer, 1992; Marting & Hammer, 1988; Dåderman & de Colli, 2014) showed that the cognitive, social, and emotional resources overlap to a certain degree, possibly because a person with positive views also has a supportive social network and is aware of emotions and able to express them. In our study, this overlap is evidenced by positive loadings of all these resources on the Efficient Coping factor.

### Modesty

Our leadership trait-based resource factor Modesty has a positive loading on honesty-humility, and at the same time, it has negative loadings on narcissism, neuroticism, and performance-based self-esteem (see Table 2 and Figure S1). In other words, Modesty is a positive resource reflecting characteristics opposite to both types of narcissism (vulnerable and grandiose), as well as opposite to traits of emotional weakness such as neuroticism, and to performance-based self-esteem. Modesty as a subscale in the HEXACO model is positively related to honesty-humility, which in turn is negatively related to counter-productive work behavior (Zettler & Hilbig, 2010). Modesty thus expresses a lack of egoism and deceitfulness and a tendency for being genuine in interpersonal relations and avoiding fraud and corruption (Lee & Ashton, 2004).

It should be noted that Modesty, like Rational Mastery, was strongly positively correlated to impression management (see Table 3), which reflects a conscious tendency towards desirable responding to the statements and items which are commonly assumed as desirable. In the case of Modesty, it means consciously attributing to oneself a tendency to be disinterested in possessing wealth, luxury goods, and signs of high social status, as well as to not feel tempted to bend the rules for personal profit.

After controlling for desirable responding and background factors, and after Bonferroni adjustment, Modesty showed a strong *negative* correlation to perceived stress (see Table 3). This finding is in line with past research on traits opposed to modesty. For example, Kajonius & Björkman (2020) showed that *vulnerable narcissism* is strongly *positively* related to perceived stress. Vulnerable narcissism differs from grandiose narcissism regarding the underlying motivation and coping flexibility (Ng et al., 2014) and uses denial for coping with stress. Fernie et al., (2016) believe that “the use of denial might be a coping response to feelings of shame when individuals with higher levels of vulnerable narcissistic traits perceive that their own needs are not being met” (p. 303). Vulnerable narcissism is also related to avoidance motivation, leading to a narrow attention scope and cognitive persistence on the current solution, indicating poor coping with stress. People high on *performance-based self-esteem* show cognitive stress symptoms (Albertsen et al., 2010), because they feel pressured to demonstrate, prove, and earn their self-worth through achievements. As Modesty is a resource reflecting the opposite characteristics, its negative correlation with perceived stress (see Table 4) is in line with theory and past research. However, Modesty is not significantly associated to task performance nor to work engagement.

### Good-Heartedness

The third resource factor comprises agreeableness, empathic concern, perspective taking, and compassionate leadership competence (emotional and spiritual). All these traits have positive factor loadings on Good-Heartedness (see Table 2 and Figure C1), indicating strong correlations of this resource factor with characteristics of these valuable human-related traits. Individual factor scores of Good-Heartedness are a weighted sum of these traits. This factor is characterized by having qualities such as communicating in a skillful way by giving feedback on the achievement of goals, being able to manage conflicts at work, controlling one’s own emotions and being less impulsive and more positive in own moods and attitudes. This kind of qualities are helpful in handling the high levels of criticism and disagreement that may arise in workplace conflicts. Leaders with such qualities are also theoretically morally good in the sense of leadership engagement, and they care about vision and values, and can see the context through holistic thinking (Ekegren & Dåderman, 2015; Ronthy, 2006, 2013). Compassionate leadership is associated with better organizational financial performance, higher follower and customer retention, and improved effectiveness and success (Cameron et al., 2011). However, Good-Heartedness, including compassionate competence, here showed nearly zero correlation with both task performance and perceived stress (see Table 3). Our regression analysis revealed a significantly negative association of Good-Heartedness with task performance, but this association was weak (see Table 4). Possibly, leaders with high factor scores of this factor perform highly in other types of individual work performance, which were not examined in the current study. We can speculate that being highly empathetic and compassionate may take capitals from other goals. Another explanation may be that Good-Heartedness contrasts correlation values those of Efficient Coping or Rational Mastery (see Table 3, correlations of these four resource factors with the two measures of socially desirable responding). We have not performed any omnibus tests for examining whether leaders with high values in Good-Heartedness differ from others by being *per se* honest, and not attempting to respond in a socially desirable way. Such examination was not aimed our study, but it could further highlight the high moral intentions of leaders with traits constituting this factor (see Table 2) while responding to our survey. The scarcity of unconscious positive bias in responses to items measuring genuine soft resources (see Table 3) may indicate that leaders with this trait-based resource did not need to protect their healthy self-esteem (Stöber et al., 2002), probably due to its already high level which is an important resource according to COR-theory. This kind of questions may be highlighted in future research.

### Rational Mastery

The fourth trait-based leadership resource factor comprises rational competence and conscientiousness. This factor is highly correlated to task performance (see Table 3) and work engagement, as well as to perceived stress. This is in line with personality theory (McCrae & Costa, 2015) which states that conscientiousness persons are self-disciplined, deliberative, achievement seeking, and motivated by their sense of duty and responsibility. These findings are consistent with meta-analyses, which found conscientiousness to be related to work performance (0.24) (O’Boyle et al., 2011), as well as to work engagement (0.39) (Young et al., 2018). To possess a high level of rational leadership competence meant performing concrete tasks that are related to the managerial assignment, such as performance against different goals. It is possible that leaders who possesses such trait-based resources are not emotionally loaded, and that empathy is not a leading star in their daily work, but that they are highly engaged in their work. For example, being high in *task performance* means “the proficiency with which individuals perform the core substantive or technical tasks central to their job” (Campbell, 1990, pp. 708–709). This factor may offer substantial survival power in stress situations, like Efficient Coping. In addition, despite a high positive correlation with self-deceptive enhancement (see Table 3) as well as impression management, this factor had a relatively strong correlation with work engagement.

Regarding our additional analyses we may conclude that female leaders had significantly higher factor scores of Efficient Coping and Good-Heartedness than male leaders, which is in line with meta-analyses (Kirkland et al., 2013; Thompson & Voyer 2014). This suggests that the identified factors are valid.

### Limitations and Suggestions for Further Research

The strength of this factor-analytical study is relatively large sample of leaders at different managerial positions from different organizations. The heterogeneous sample increases variation, making factor analyses more reliable. Below, we will discuss some limitations of our study. First, future research could test whether self-reported trait-based leadership resources are congruent with other data, for example with the followers’ perceptions of their leaders’ levels of task performance, perceived stress, or work engagement. However, this may be difficult to assess properly. There are possible discrepancies between leaders’ perception of their level of, for example, trait EI, and how that level is described by their followers. Such a study would be time-consuming and costly, and we had no opportunity to perform such data sampling. The fact that all our measures were self-reported can also be seen as a limitation.

Second, it could be beneficial to design a longitudinal study to draw causal conclusions from the data. However, such a study may be difficult to implement due to the leaders’ limited time to fill in the questionnaires needed to make such a follow-up study meaningful. It might also become contaminated with severe drop-out and ethical issues due to the necessity of keeping some vulnerable information (e.g., exact identities) intact. However, despite its cross-sectional design, the current study has a high response rate (73%), making it possible to draw valid conclusions.

Third, self-report biases may have contributed to errors in the measurement of trait-based leadership resource factors that are generally considered to be positive, although an attempt was made to control for social desirability bias. Specifically, self-report measures are vulnerable to social desirability bias resulting from, for example, narcissists whose “exaggerated self-appraisal may be inflated or deflated, or vacillate between extremes” (American Psychiatric Association, 2013). Narcissists possess a high lie-telling ability (Zvi & Elaad, 2018) and a high need for self-enhancement and superiority (Campbell et al., 2011), especially regarding intelligence, extraversion, and their (believed) leadership competence (Grijalva & Zhang, 2016). Table 3 shows that two of the trait-based leadership resource factors (Modesty and Rational Mastery) showed the largest values of correlation coefficients in relation to impression management. Impression management was positively related to Modesty, which may be expected because traits in Modesty (e.g., being honest) are socially desirable, not only in a leader. Both impression management and self-deceptive enhancement were positively related to Rational Mastery, which may be explained by a conscious as well as unconscious drive to appear as a task performance-oriented leader in the own and others’ eyes.

Fourth, we limited our research questions regarding work performance to *task* performance only and did not analyze other aspects of individual work performance, such as contextual performance and counterproductive work behavior (Koopmans et al., 2011, 2013) or adaptive performance (Griffin et al., 2007; Stasielowicz, 2020). Future research may relate the four trait-based leadership resource factors to other aspects of individual work performance, because leaders’ behaviors may require focus on both task-focused (such as transactional behaviors, initiating structure, boundary spanning) and person-focused (such as transformational behaviors, consideration, empowerment, motivational behaviors) forms of leadership (Burke et al., 2006). Other forms of work performance may be especially important for exploring the relationship with the Good-Heartedness trait-based resource factor, which mirrors valuable human-related traits as agreeableness, empathic concern, perspective taking, and compassionate leadership competence. US president Theodore Roosevelt once remarked, “Softness of heart is an admirable quality, but when it extends its area until it also becomes softness of head, its results are anything but admirable. It is a good thing to combine a warm heart with a cool head” (Roosevelt, 1900), and future research may have specific focus on individual differences of this resource.

Lastly, our CFA is conducted on the same data as EFA, and Figure S1 serves illustrative purposes only. The data set of 344 managers was too small for conducting EFA on one half of the data and CFA on the other. Having in mind the complexity of trait-based leader resources (Nielsen et al., 2017; Zaccaro, 2007), and that “personality trait inventories often perform poorly when their structure is evaluated with confirmatory factor analysis (CFA)” (Hopwood & Donnellan, 2010, p. 332), we recommend the use of such a procedure in forthcoming studies.

## Conclusions

Considered together, we were able to differentiate the four resource factors and their relationships to task performance, perceived stress, and work engagement, which can have practical implications for the recruitment of leaders. We could show that the same managerial qualities are good for the result of work in different types of organizations.

Organizations should be mindful of their leaders’ levels of common traits and resources, so that they may develop appropriate leadership training. This could stimulate leaders to obtain, retain, gain, and protect valuable trait-based resources in the context of their complex leadership roles.

## Electronic Supplementary Material

Below is the link to the electronic supplementary material.

Supplementary Material 1

Supplementary Material 2

## Data Availability

The data generated and analyzed during this study, except the participants’ background information, are included in this published article (see its electronic SPSS-data file as Supplementary material CUPS-N344).

## Footnotes

1 In 708 meta-analytically derived correlations from 87 meta-analyses, “which were relevant to the association between two conceptually distinct constructs” (Gignac & Szodorai, 2016, p. 75), Gignac and Szodorai found that the 25th, 50th, and 75th percentiles corresponded to correlations equal to 0.11, 0.19., and 0.29; only 2.7% of the correlations were 0.50 or greater. They therefore recommended that individual differences researchers consider correlation values of 0.10, 0.20, and 0.30 to be small, typical (medium*)*, and large, respectively (see Gignac & Szodorai 2016, p. 75, and Table 1, left-hand side). We have applied these recommendations in the assessment of our results.
