# Supplementary material for: Leading with a cool head and a warm heart: trait-based leadership resources linked to task performance, perceived stress, and work engagement
Source: Curr Psychol. 2022 Nov 21:1–22. Online ahead of print. doi: 10.1007/s12144-022-03767-8 (PMC9684842; doi:10.1007/s12144-022-03767-8)
Supplement: Supplementary file 2 — Supplementary Material 2 [file 12144_2022_3767_MOESM2_ESM.docx]

**Leading with a cool head and a warm heart: Trait-based leadership resources linked to task performance, perceived stress, and work engagement**

**Supplementary material**

**Table S1**

*Pearson correlations between scales included in the factor analyses*

| **Scale** | **Com** | **EI** | **PT** | **EC** | **VNarc** | **GNarc** | **SE** | **COG** | **SOC** | **EMO** | **SP** | **PHY** | **E** | **A** | **C** | **N** | **O** | **H-H** |
| --- | --- | --- | --- | --- | --- | --- | --- | --- | --- | --- | --- | --- | --- | --- | --- | --- | --- | --- |
| EI | .42 |  |  |  |  |  |  |  |  |  |  |  |  |  |  |  |  |  |
| PT | .47 | .47 |  |  |  |  |  |  |  |  |  |  |  |  |  |  |  |  |
| EC | .36 | .28 | .38 |  |  |  |  |  |  |  |  |  |  |  |  |  |  |  |
| VNarc | -.20 | -.50 | -.38 | -.19 |  |  |  |  |  |  |  |  |  |  |  |  |  |  |
| GNarc | .17 | .09 | -.08 | -.03 | .21 |  |  |  |  |  |  |  |  |  |  |  |  |  |
| SE | .02 | -.21 | -.13 | -.01 | .44 | .29 |  |  |  |  |  |  |  |  |  |  |  |  |
| COG | .28 | .52 | .24 | .10 | -.31 | .18 | -.20 |  |  |  |  |  |  |  |  |  |  |  |
| SOC | .42 | .59 | .37 | .27 | -.45 | .11 | -.14 | .64 |  |  |  |  |  |  |  |  |  |  |
| EMO | .35 | .49 | .36 | .25 | -.26 | .12 | -.15 | .64 | .69 |  |  |  |  |  |  |  |  |  |
| SP | .26 | .39 | .31 | .15 | -.18 | -.01 | -.06 | .41 | .44 | .51 |  |  |  |  |  |  |  |  |
| PHY | .06 | .27 | .03 | -.09 | -.20 | .03 | -.21 | .35 | .31 | .26 | .19 |  |  |  |  |  |  |  |
| E | .22 | .38 | .11 | .13 | -.20 | .37 | -.04 | .31 | .43 | .29 | .05 | .13 |  |  |  |  |  |  |
| A | .44 | .44 | .50 | .56 | -.29 | .01 | -.01 | .16 | .47 | .39 | .24 | -.03 | .27 |  |  |  |  |  |
| C | .14 | .29 | .08 | .04 | -.28 | .03 | -.15 | .24 | .18 | .17 | .15 | .28 | .07 | .08 |  |  |  |  |
| N | -.13 | -.43 | -24 | .02 | .42 | .12 | .35 | -.33 | -.27 | -.22 | -.15 | -.30 | -.09 | -.04 | -.22 |  |  |  |
| O | .23 | .35 | .27 | .12 | -.15 | -.02 | -.01 | .23 | .27 | .27 | .19 | .11 | .23 | .30 | -.03 | -.11 |  |  |
| H-H | .14 | .26 | .35 | .20 | -.39 | -.45 | -.29 | .12 | .28 | .22 | .20 | .14 | .01 | .29 | .03 | -.27 | .19 |  |
| RQ | .46 | .32 | .18 | .18 | -.25 | .11 | -.05 | .15 | .24 | .18 | .15 | .19 | .15 | .21 | .43 | -.09 | .09 | .06 |

Note: *N* = 344. See the *Materials and Methods* for the abbreviations of the instruments. Com = Compassionate leadership competence (LIQ3). EI = Global trait emotional intelligence (TEIQue-SF). PT = Perspective taking (IRI). EC = Empathic concern (IRI). VNarc = Vulnerable narcissism (HSNS). GNarc = Grandiose narcissism (SD3). SE = Performance-based self-esteem (PBSE). COG = Cognitive coping resource (CRI). SOC = Social coping resource (CRI). EMO = Emotional coping resource (CRI). SP = Spiritual/Philosophical coping resource (CRI). PHY = Physical coping resource (CRI). E = Extraversion (Mini-IPIP6). A = Agreeableness (Mini-IPIP6). C = Conscientiousness (Mini-IPIP6). N = Neuroticism (Mini-IPIP6). O = Openness to experience (Mini-IPIP6). H-H = Honesty-Humility (Mini-IPIP6). RQ = Rational leadership competence (LIQ3).

**Figure S1**

*Confirmatory factor analytic model tested for the trait-based leadership resource factors from exploratory factor analysis presented in Table 2*


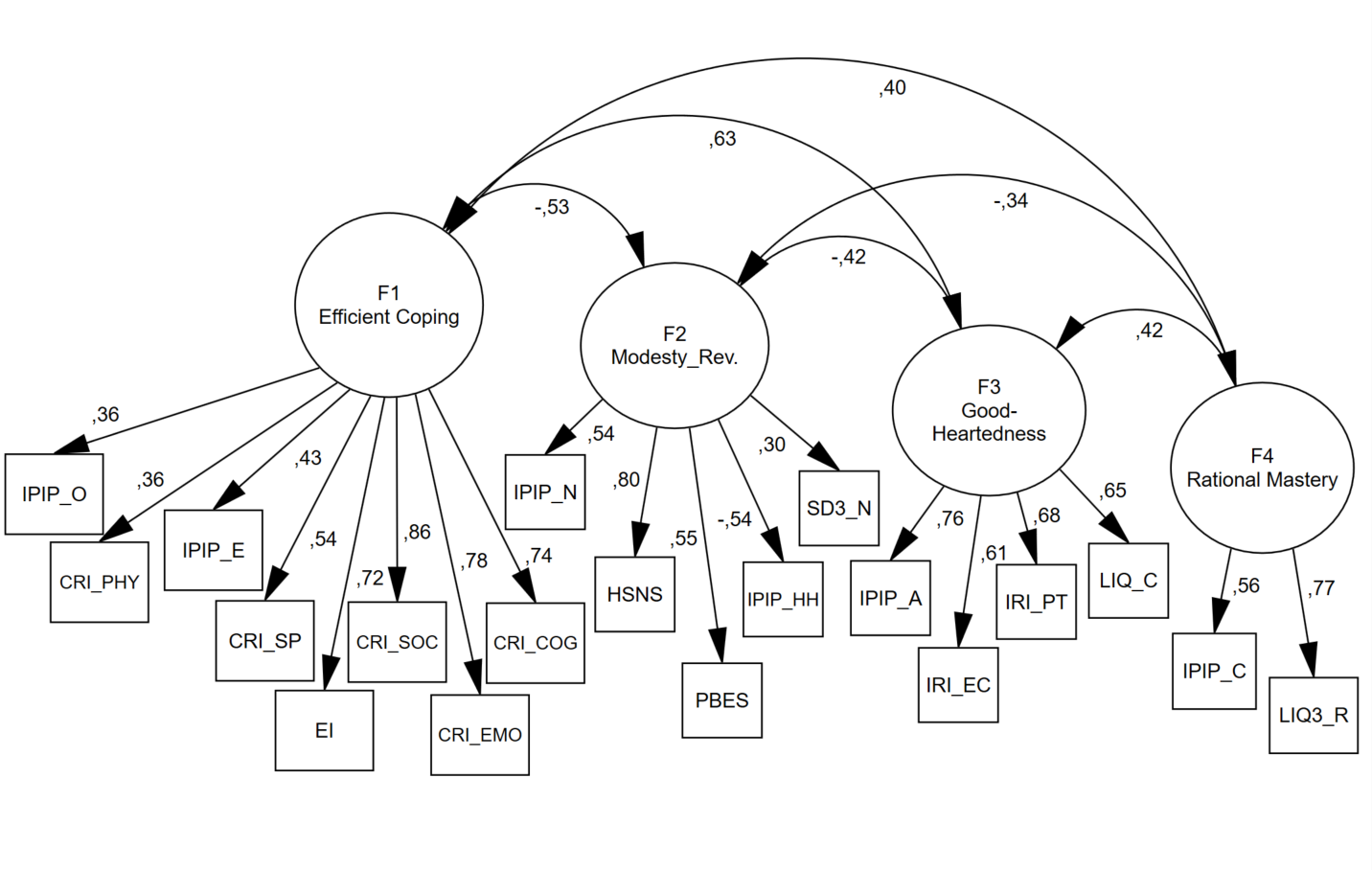


Note: Loading signs of Factor 2 (Modesty) are reversed to reflect a resource. Chi-square (146) was 705 (*p* < .001), and the root mean square error of approximation (RMSEA) was .106 [.098, .114]. The value of RMSEA was similar to those values that have been typically observed for well-known complex personality inventories with documented evidence of criterion validity, among others NEO-PI-R (see Hopwood & Donnellan, 2010, Table 1).

**Reference**

Hopwood, C. J., & Donnellan, M. B. (2010). How should the internal structure of personality inventories be evaluated? *Personality and Social Psychology Review, 14,* 332–346.
